# Supplementary material for: Mechanical ventilation in acute myocardial infarction: Outcomes from a prospective audit at a cardiovascular hospital in Pakistan
Source: PLoS One. 2023 Aug 18;18(8):e0290399. doi: 10.1371/journal.pone.0290399 (PMC10437963; doi:10.1371/journal.pone.0290399)
Supplement: Attachment — Submitted filename: Response to Reviewers.docx [file pone.0290399.s002.docx]

**Dear Editor,**

Thank you for sharing valuable feedback of reviewers regarding our submission to the PLOS ONE titled **“Mechanical Ventilation in Acute Myocardial Infarction: Outcomes from a Prospective Audit at a Cardiovascular Hospital in Pakistan”**. Manuscript ID: [PONE-D-23-18200] - [EMID:24ddb4a8281ed9f6]

We have revised the manuscript as per the specific comments as listed below. We believe these changes have strengthened the quality of our manuscript and that you will find it suitable for publication in the Journal.

**Reviewer 1
Comment** 1- A flow chart is needed.

**Response:** Thank you for the comment. As suggested, a patient selection flow chart is added to the manuscript.

**Comment** 2- The calculation of the minimal sample size needed to conduct the study should be demonstrated in the methodology.

**Response:** Thank you for the comment. As suggested, details regarding consideration for the minimum sample size are added in the methods section.

**Comment** 3- The authors better to determine the types of myocardial infarction included, if they enrolled all MI types, then better to define MI in the methodology.

**Response:** Thank you for the comment. As suggested, details regarding the categorization of MI are added to the methods section.

**Comment** 4- The authors should improve the coherence of the introduction. Frailty is an independent topic which might not be suitable to analyse here.

**Response:** Thank you for the comment. As suggested, frailty has been omitted from the introduction and this section has been improved for the flow.

**Comment** 5- The discussion should be re written in a more academic manner. Start the first paragraph with the main findings you like highlight, then proper critical appraisal in the light of prior literatures in the subsequent paragraphs.

**Response:** Thank you for the comment. As suggested, we have added study highlights in the first paragraph and a discussion of the findings with reference to the literature in subsequent paragraphs.

**Comment** 6- In a paragraph before the limitations, highlight the clinical implications of the study in a clear and practical manner.

**Response:** Thank you for the comment. As suggested, we have added the clinical implications of the study before the limitation section.

**Comment** 7- All the potential limitations should be highlighted properly and in acceptable order in the limitations section.

**Response:** Thank you for the comment. As suggested, we have elaborated on the limitation section.

**Comment** 8- Improve the quality and resolution of the figures.

**Response:** Thank you for the comment. As suggested we have improved the resolution of figures

**Comment** 9- Describe the abbreviations once first mentioned. In the abstract; IABP. In the text; GCS, RSBI, etc...

**Response:** Thank you for pointing it out. As suggested, we have added full forms of all the abbreviations at the first instance

**Reviewer 2
Comment** The importance for the readership is explicitly justified. One concrete aim are formulated

The literature search is described in details, including search terms and inclusion criteria.

Key statement are supported by references.

Appropriate evidence is generally presented

**Response:** Thank you for the kind appreciation and acknowledgment of the work

**Reviewer 3
Comment** 1- Other diseased vessel are not clear if significant lesions and need further revascularization or not.

**Response:** Thank you for the comment. However, the focus of the study was the audit of ICU care but stage PCI and other management strategies for other diseased vessels may have an impact on short-term outcomes, unfortunately, we had not documented this information in our audit and it has been acknowledged in the limitation section.

**Comment** 2-Noninvasive mechanical ventilation still a good choice in acute cardiac insult if patient can cope and not mentioned in your study.

**Response:** Thank you for the comment. However, our inclusion criteria were explicitly those patients who required invasive mechanical ventilation after AMI.

**Comment** 3- no comments on mechanical complications as rupture chordae and papillary muscle dysfunction as a cause of MR .

**Response:** Thank you for pointing it out. We have document MR based on departmental ECHO but unfortunately, the cause of MR was not documented in this audit.

**Comment** 4- the need of glycoprotein IIB IIIA , enoxaparin.UFH are not mentioned as affecting the bleeding risk.

**Response:** Thank you for pointing it out. All patients were managed as per the standard clinical practice guidelines. Details are added in the methods section.

**Comment** 5- The indication of RRT should be clarified (anuria, metabolic acidosis or volume overload

**Response:** Thank you for the comment. As suggested we have added indications for the RRT in our methods section.
